# Chitosan modifies glycemic levels in people with metabolic syndrome and related disorders: meta-analysis with trial sequential analysis

**DOI:** 10.1186/s12937-020-00647-4

**Published:** 2020-12-01

**Authors:** Wenfang Guo, Letai Yi, Baochang Zhou, Minhui Li

**Affiliations:** 1 Inner Mongolia Autonomous Region Academy of Traditional Medicine, No. 11 Jian Kang Street, Hohhot, 010020 Inner Mongolia China; 2 Inner Mongolia Hospital of traditional Chinese Medicine, Hohhot, 010020 China; 3 Inner Mongolia Medical University, Hohhot, 010110 China; 4 Baotou Medical College, Baotou, 014060 China

**Keywords:** Chitosan, Glucose, Insulin, HbA1c, Meta-analysis, Trial sequential analysis

## Abstract

**Background:**

Chitosan supplementation has been shown to modulate glycemic levels; however, studies have reported conflicting results. The present meta-analysis with trial sequential analysis was conducted to verify the overall influence of chitosan on glycemic levels in patients with metabolic syndrome.

**Methods:**

The PubMed, Cochrane library, and EMBASE databases were systematically searched for randomized controlled studies of chitosan intake and glycemic levels.

**Results:**

A total of ten clinical trials including 1473 subjects were included in this meta-analysis. Pooled effect sizes were determined by random-effects meta-analysis. Subgroup analysis was performed to analyze the sources of heterogeneity and their influence on the overall results. The results revealed a significant reduction in fasting glucose levels (SMD: − 0.39 mmol/L, 95% CI: − 0.62 to − 0.16) and hemoglobin A1c (HbA1c) levels (SMD: -1.10; 95% CI: − 2.15 to − 0.06) following chitosan supplementation but no effect on insulin levels (SMD: − 0.20 pmol/L, 95% CI: − 0.64 to 0.24). Subgroup analyses further demonstrated significant reductions in fasting glucose levels in subjects administered 1.6–3 g of chitosan per day and in studies longer than 13 weeks. Trial sequential analysis of the pooled results of the hypoglycemic effect demonstrated that the cumulative Z-curve crossed both the conventional boundary and trial sequential monitoring boundary for glucose and HbA1c.

**Conclusions:**

The glucose level of patients who are diabetic and obese/overweight can be improved by supplementation with chitosan for at least 13 weeks at 1.6–3 g per day. Additional clinical research data are needed to confirm the role of chitosan, particularly in regulating glycosylated hemoglobin and insulin.

**Supplementary Information:**

The online version contains supplementary material available at 10.1186/s12937-020-00647-4.

## Background

Metabolic syndrome (MetS) represents a group of metabolic disorders characterized by elevated insulin resistance and blood glucose concentrations, as well as hypertension, atherogenic dyslipidemia, and obesity [1]; these factors lead to an increased risk of cardiovascular disease, type 2 diabetes mellitus, and other all-cause mortality [1, 2]. Globally, the prevalence of MetS has been increasing annually and has become a major medical and public health problem in the last few decades [2, 3]. The prevalence of MetS in the United States is estimated to be 34% [4]. In Iran, based on the Tehran Lipid and Glucose Study, the prevalence of MetS was 42 and 24% in adult men and women, respectively [5]. Furthermore, the overall incidence of MetS in adults aged 20 years and above was reported at 550.9 per 10,000 individuals: in men, it the rate was 794.2 per 10,000 and in women was 443.5 per 10,000 individuals [6]. The prevalence of MetS in Korea has increased continuously from 24.9% in 1998 to 29.2% in 2001, 30.4% in 2005, and 31.3% in 2007 [7]. The number of patients with diabetes has reached more than 425 million, which is expected to increase to 629 million in the next 30 years without appropriate intervention. Moreover, the 352 million people with impaired glucose tolerance are expected to ultimately develop diabetes in the near future [8, 9]. The prevalence of MetS in patients with type 2 diabetes mellitus has been higher. For instance, this rate has been reported as 57% in India [10], 70.4% in Iran [11], 68.6% in Ghana [12], and 70.1% in Ethiopia [13]. In addition to the general increase in morbidity in these populations, associated medical expenses have imposed a heavy economic burden, thereby diverting resources from other aspects of public health and social and economic development. Given the lack of sufficient funds for preventing MetS [8, 9], adoption of a fiber-rich diet has emerged as an adjuvant treatment for hyperglycemia [14, 15]. Dietary fiber regulates energy intake by prolonging chewing time, increasing satiety, reducing calorie density, and delaying gastric emptying [16, 17]. In addition, dietary fiber exerts hypoglycemic effects in patients with type 2 diabetes based on a meta-analysis of 11 randomized controlled trials (RCTs) [18].

As a deacetylated form of chitin, chitosan is an important dietary fiber source and among the most abundant natural polysaccharides worldwide [19], as it is derived from numerous taxa, including crustacean shells, insect exoskeletons, parasitic nematode eggs and gut linings, and fungi cell walls [20]. Chitosan is generally considered as a non-toxic nutritional cellulose supplement [21], and several animal studies and human clinical trials have demonstrated its good health effects, such as the prevention of cancer, hypertension, hyperglycemia, diabetes, dyslipidemia, and inflammation [22–26].

It is generally accepted that high-fiber foods are beneficial for managing MetS such as type 2 diabetes mellitus and/or hyperglycemia [27]. Most authorities recommend a higher dietary fiber intake for such patients than that recommended for the general population [28]. Several hundred tons of dietary chitosan products are consumed annually in Europe and the USA [29]. Chitosan can play a hypoglycemic effect without decreasing the appetite in induced Type 2 diabetes rats on a high-fat high-sugar diet and low dose streptozotocin [30]. Further, chitosan has been reported to improve blood lipid levels, blood glucose levels, and body weight in patients with MetS without dietary intervention [29, 31–34]. Notably, chitosan oligosaccharides have been shown to promote the proliferation of β-cells and the recovery of damaged β-cell functions which can produce insulin and thus increases insulin sensitivity [33, 35]. A study also have shown that chitosan can improve lipid metabolism related to diabetes [36]. Many researchers have focused on the combined, synergistic, or additive effects of these molecules to treat this complex metabolic disorder [35–38].

Although the potential hypoglycemic effect of chitosan has been evaluated in clinical trials, these studies substantially vary with respect to the research design, population characteristics, chitosan supplement dosage, and duration, leading to mixed results and conclusions [29, 31–34, 37, 39–43]. No systematic review or meta-analysis has been conducted to evaluate the effect of chitosan on human glycemic levels. Therefore, in the present study, we conducted a comprehensive meta-analysis of all available RCTs with trial sequential analysis to evaluate the effects of chitosan on glycemic levels in adults. These results provide a basis and improved evidence for the use of chitosan supplementation in the prevention, treatment, and management of MetS.

## Methods

### Search strategy and study selection

This review was conducted according to the guidelines of the Preferred Reporting Items for Systematic Reviews and Meta-Analyses (PRISMA) [44]. PubMed, EMBASE, and Cochrane Library databases were searched through September 7, 2020. We used the following Medical Subject Headings (MeSH) and corresponding keywords: “chitosan” OR “chitin” OR “poliglusam” OR “polyglucosamine” OR “chitosan oligosaccharide”. Our search was limited to English-language publications and clinical investigations conducted in human subjects. To identify potentially any other missing RCT, we further scrutinized the reference citations of relevant review articles. The search strategy are shown in Supplementary file 1. All RCTs meeting the following criteria were included in the analysis: 1) human subjects with either a parallel or crossover design; 2) the study assessed the impact of chitosan or chitosan supplements; 3) placebo was used as control; 4) the study assessed fasting plasma glucose, insulin, hemoglobin A1c (HbA1c) as outcome measures; and 5) patients with MetS, such as dyslipidemia, obesity, and diabetes. Exclusion criteria were as follows: 1) reviews or meta-analyses; 2) chitosan in combination with other supplements or interventions; and 3) lack of data for the standard deviation for the intervention and control groups.

The preliminary selected studies were imported into Endnote × 9 software to remove repetitive literature and were then manually screened for the article title and abstract. Two reviewers screened the results of literature retrieval and retrieved the eligible abstracts. The full text was further screened, and data reliability was evaluated; any disagreements were resolved by discussion based on valid reference between two authors and a third investigator.

### Quality assessment

Two investigators independently used the Cochrane’s risk of bias tool [45] to systematically review the interventions for assessing the quality of included trials. Assessed factors included sequence generation, allocation concealment, blinding of participants and personnel, blinding of outcomes, incomplete outcome data, and selective reporting. Articles were judged to be of high, low, or unclear quality based on the following: randomized sequence generation, allocation concealment, blinding of participants and study personnel, blinding of outcome assessors, incomplete outcome data, selective reporting, and other biases.

### Data extraction and management

The major demographic and clinical data from each selected study were screened and extracted independently by two investigators using a pre-designed Excel sheet. The following information was extracted: first author’s name, year of publication, study location, sample size, recruitment methods, study design details, intervention duration (in weeks), participants’ mean age, chitosan dosage and reported outcomes. When parallel trials included in our analysis had multiple intervention arms, the outcomes of all arms were extracted if they met the study criteria; the data of all experimental arms were grouped, and the pooled effect size was compared with that of the control group in an overall meta-analysis. Glucose levels were collated in mmol/L (1 mmol/L = 18 mg/dL) and insulin levels were collated in pmol/L (1 pmol/L = 6.965 mIU/L).

### Data synthesis and meta-analysis

Statistical analyses were performed using STATA version 12.0 and RevMan software (version 5.3 for windows). Statistical heterogeneity was assessed by using the I^2^ statistic and Cochran (Q) test [46]; I^2^ values of 25, 50, and 75% indicated low, moderate, and high heterogeneity, respectively. When significant heterogeneity was determined (*p* < 0.05 or I^2^ > 50%), the random-effects model was used to determine the overall effect size; otherwise, the fixed-effects model was used. The pooled effect of chitosan intervention was determined using the inverse variance method to estimate the standardized mean difference (SMD) and 95% confidence interval (CI). Subgroup analysis was performed to identify probable sources of heterogeneity. Egger’s regression test was used to assess the potential for publication bias [47]. Trial sequential analysis was conducted to evaluate the accumulated evidence with TSA version 0.9.

## Results

### Study selection

**Fig. 1:**
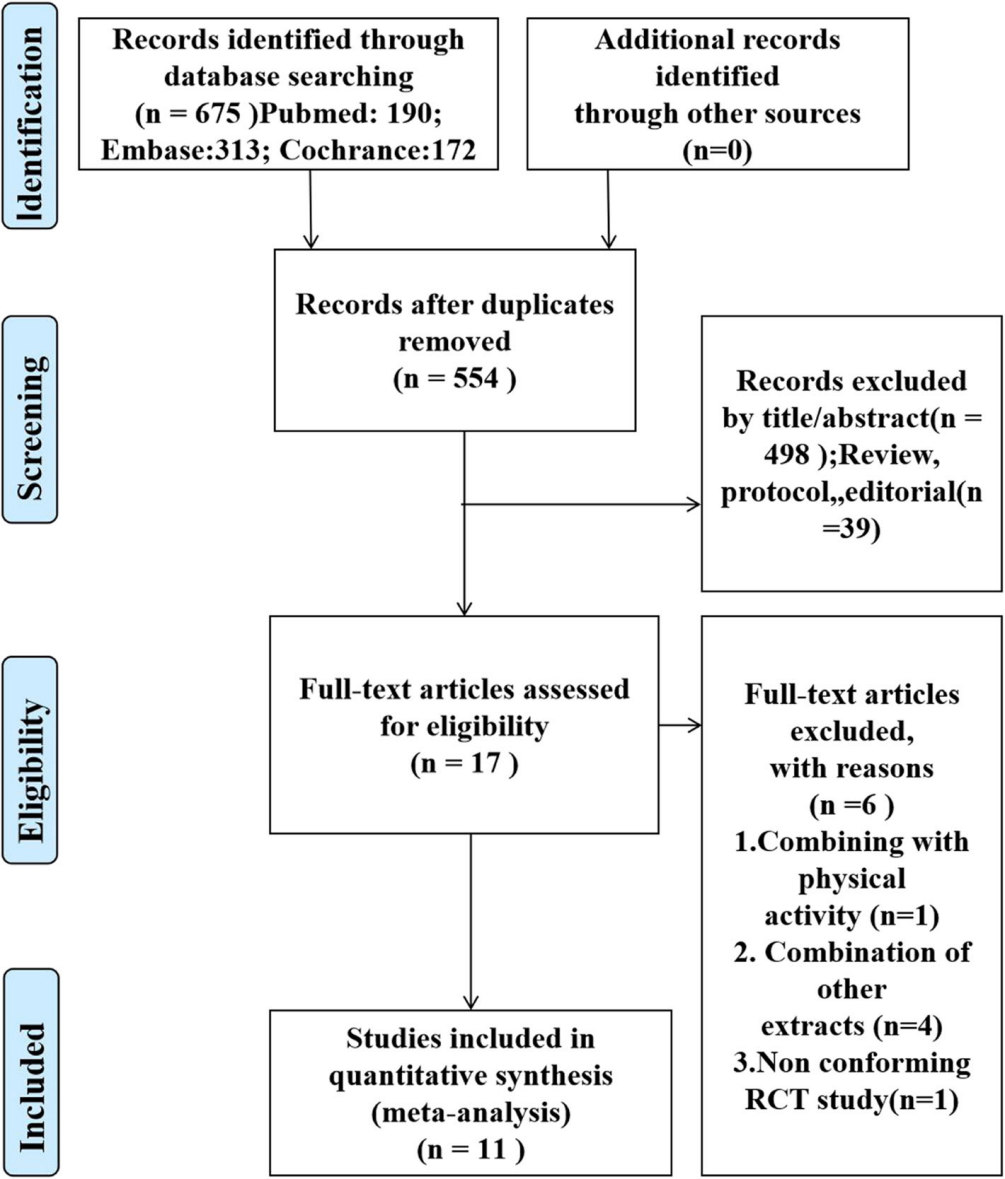
PRISMA flow-diagram of the study selection process

Figure 1 shows the flow chart of the study selection process. An initial search identified 675 potential articles, 121 of which were found to be duplicates, leaving 554 articles in the first stage of screening. After excluding non-relevant studies according to the title and abstract, 17 articles were selected for the next stage of full-text screening. One article [48] did not specify the inclusion criteria, such as whether the study population had MetS or if a random distribution and blinding method were used. One study combined chitosan supplementation with physical activity [49], and four studies combined chitosan with other extracts (containing gymnemic acid, mulberry leaf extract, green tea extract, chitosan, and kidney bean extract) [50–53]. Finally, 11 studies met the inclusion criteria and were included in the current study.

### Test for publication bias

Egger’s regression test was performed to detect publication bias among the studies included in the meta-analysis. The test showed no evidence of significant publication bias when evaluating the effect of chitosan on fasting glucose levels (t = − 1.28, *p* = 0.225).

### Study characteristics

The publications included in the meta-analysis are described in Table 1. Of the 11 included articles, three used a cross-over [29, 37, 40] design and others used a parallel design [31–34, 39, 41–43]. Trials were conducted in China [41], Italy [31], India [42], Korea [33, 37], USA [39], Mexico [32], Finn [29], New Zealand [34], Finland [40], and Singapore [43]. These studies were published between 2001 and 2019. Sample sizes ranged from 6 to 118, and intervention durations ranged from 6 to 51 weeks. In total, 1473 participants (749 in the intervention group and 724 in the control group) were included in the final analysis. Subjects in five articles [31, 32, 34, 42, 43] were overweight or obese; three articles [29, 39, 40] included patients with dyslipidemia, and three articles [33, 37, 41] evaluated patients with prediabetes or diabetes. The mean age of the patients was 25–69 years. Compliance measures are not clearly stated in these studies. The recruitment methods of participants include: sending invitation letters to the initially selected people [29, 40]; advertising or newspapers [33, 34, 39, 43]; recruiting from specific hospitals, laboratories [37], and there were four articles that did not specify the way of recruitment [31, 32, 41, 42]. Of these studies, 9 trials reported the glucose change as an outcome measure [29, 31–34, 37, 39–41], 3 articles reported insulin change [33, 39, 43], 3 articles reported ported HbA1c [33, 41, 42]. Nine of the 11 articles used a randomized double-blind placebo-controlled approach.

**Table 1:** Characteristics of included studies

Authors (Ref)	Publication year	Age mean (SD) control/intervention	Sample size (control/intervention)	Country/population	Study design	Intervention (name and daily dose)	Duration (weeks)	Presented data	Participant recruitment methods	Results
Zhao [41]	2017	67.8 (7.5) / 68.5 (8.0)	50 / 50	China / diabetes	Parallel	0.1 g/d	42 weeks	Glucose and HbA1c	Not stated	Decrease
Cornelli-3mon [31]	2017	47.0 (3.75) / 46.4 (4.42)	48 / 49	Italy / obese	Parallel	1.6 g/d	13 weeks (3 month)	Glucose	Not stated	Decrease
Cornelli-6mon [31]	2017	47.0 (3.75) / 46.4 (4.42)	48 / 49	Italy / obese	Parallel	1.6 g/d	26 weeks (6 month)	Glucose	Not stated	Decrease
Cornelli-9mon [31]	2017	47.0 (3.75) / 46.4 (4.42)	48 / 49	Italy / obese	Parallel	1.6 g/d	38 weeks (9 month)	Glucose	Not stated	Decrease
Cornelli-12mon [31]	2017	47.0 (3.75) / 46.4 (4.42)	48 / 49	Italy / obese	Parallel	1.6 g/d	51 weeks (12 month)	Glucose	Not stated	No effect
Trivedi-90 day [42]	2016	36.3(10.5)/35.5(11.2)	64/32	India / overweight and obese	Parallel	2.5 g/d	12 weeks	HbA1c	Not stated	Decrease
Trivedi-45 day [42]	2016	36.3(10.5)/35.5(11.2)	64/32	India / overweight and obese	Parallel	2.5 g/d	6 weeks	HbA1c	Not stated	No effect
Kim [33]	2014	54.4 (2.02) / 57.8 (1.78)	26 / 25	Korea / prediabetes	Parallel	1.5 g/d	12 weeks	Glucose, Insulin and HbA1c	Recruited from Hospital and advertisement in local newspapers	Decrease:Glucose and HbAIc;No effect: Insulin
Bays-4.5 g [39]	2013	49.1 (11.2) / 52.0 (11.3)	35 / 33	USA / dyslipidemia	Parallel	4.5 g/d	6 weeks	Glucose and Insulin	Recruited in response to advertisements or from study site databases	No effect
Bays-1.5 g [39]	2013	50.9 (10.3) / 52.0 (11.3)	35 / 32	USA / dyslipidemia	Parallel	1.5 g/d	6 week	Glucose and Insulin	Recruited in response to advertisements or from study site databases	No effect
Hernández-González [32]	2010	46.1 (6.3) /41.6 (6.3)	6月6日	Mexico / obese	Parallel	2.25 g/d	13 weeks	Glucose	Not stated	No effect
Lehtimäki-carrier [29]	2005	43.5 (8.8) / 43.9 (8.9)	29 / 29	Finn / hypercholesterolaemic	Crossover	2.4 g/d	13 weeks	Glucose	Invitation letters	No effect
Lehtimäki-noncarrier [29]	2005	44.8 (7.7) / 45.0 (7.8)	55 / 55	Finn/hypercholesterolaemic	Crossover	2.4 g/d	13 weeks	Glucose	Invitation letters	No effect
Mhurchu [34]	2004	47 (11.7) / 48 (11.5)	116 / 118	New Zealand / overweight and obese	Parallel	3 g/d	24 weeks	Glucose	Recruited using newspaper advertisements	Decrease
Metso [40]	2003	46 (8)	90 / 93	Finland / hypercholesterolaemic	Crossover	1.2 g/d	13 weeks	Glucose	Invitation letters	No effect
Ho [43]	2001	43.3(7.7) / 42.6(6.7)	32/36	Singapore/Obesity	Parallel	0.25 g/d	16 weeks	Insulin	Recruited from the general public by advertisement	No effect
Jeong [37]	2019	28.6(1.49)/28.6(1.49)	19/19	Korea/prediabetes	Crossover	0.25 g/d	2 weeks	Glucose	Recruited from the Clinical Nutrigenetics/Nutrigenomics Laboratory at Yonsei University	Decrease

### Quality assessment and potential bias

**Fig. 2:**
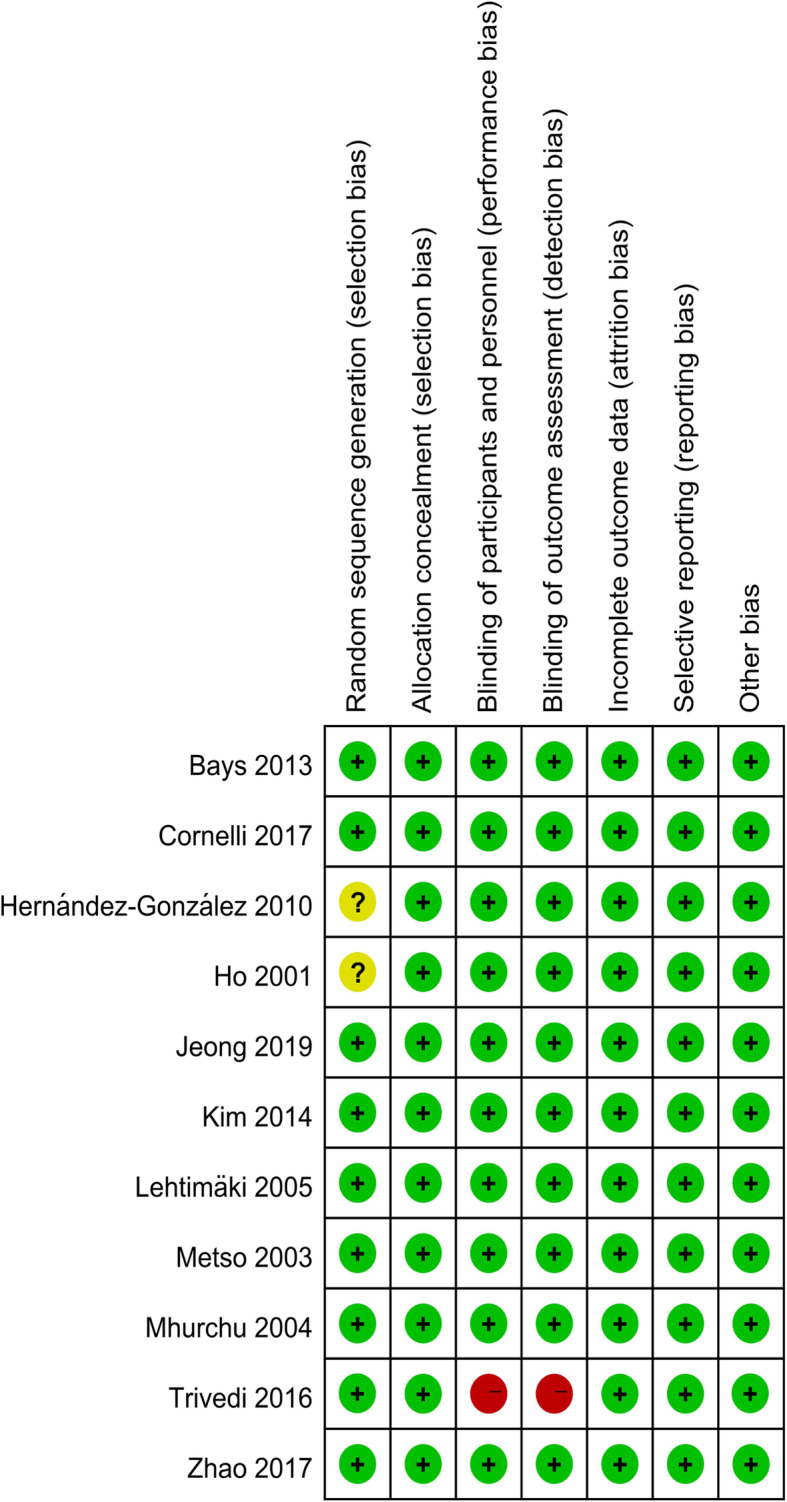
Summary of authors’ judgments about each risk of bias item for each included study

The details risk-of-bias summary is shown in Fig. 2. All included studies adopted randomization criteria, although two [32, 43] did not clearly delineate the process of random sequence generation. Overall, 10 trials were considered to have a low risk of bias and one was considered to have a high risk of bias [42].

### Effects of chitosan on glucose, insulin, and HbA1c levels

**Fig. 3:**
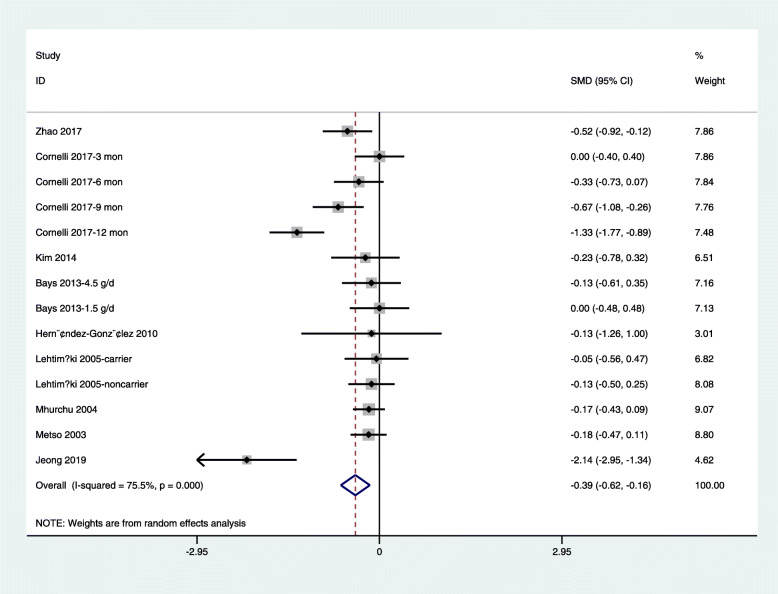
Forest plot detailing weighted mean difference and 95% confidence intervals for the impact of chitosan supplementation on fasting glucose levels

**Fig. 4:**
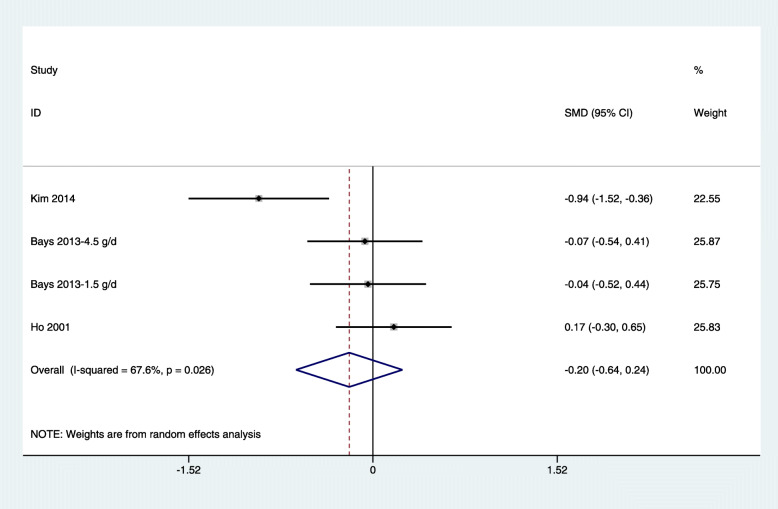
Forest plot detailing weighted mean difference and 95% confidence intervals for the impact of chitosan supplementation on insulin levels

**Fig. 5:**
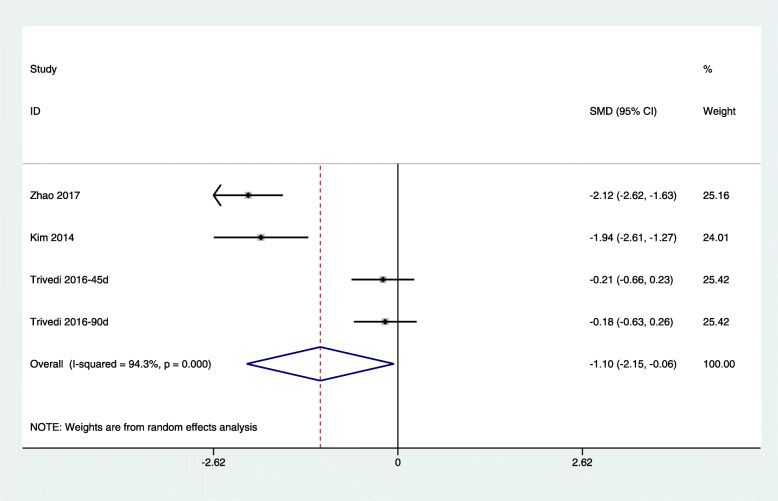
Forest plot detailing weighted mean difference and 95% confidence intervals for the impact of chitosan supplementation on HbA1C levels

**Fig. 6:**
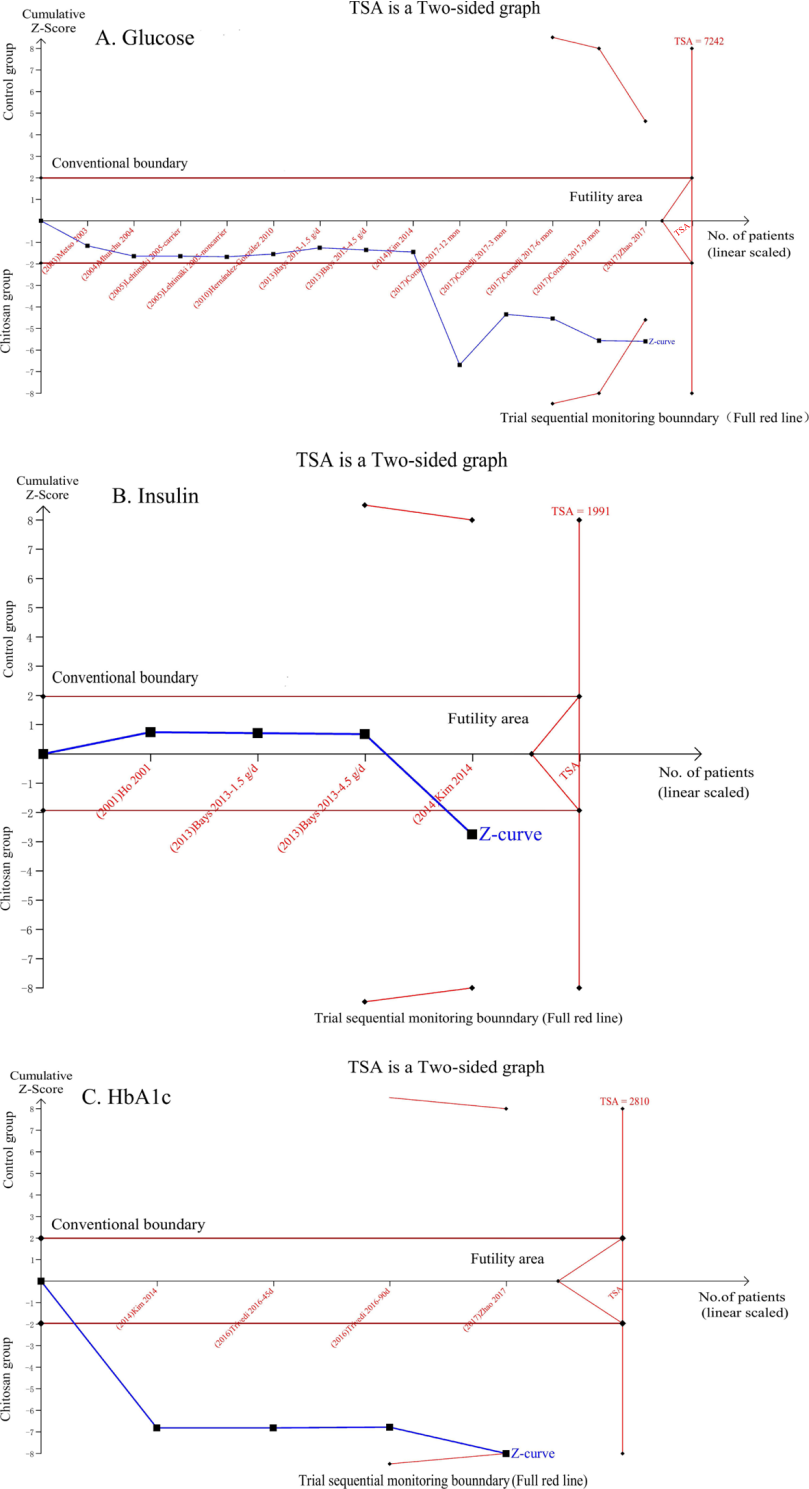
Trial sequential analysis for the impact of chitosan supplementation on glycemic levels. **a** TSA on pooled result of glucose: The cumulative sample size was over the required information size (RIS) of 7242 and cumulative Z-curve crossed both the trial sequential monitoring boundary and conventional boundary. **b** TSA on pooled result of insulin: the cumulative Z-curve was crossed conventional boundary but not crossed the trial sequential monitoring boundary. **c** TSA on pooled result of HbA1c: the cumulative sample size was over the RIS of 2810 and cumulative Z-curve crossed both the trial sequential monitoring boundary and conventional boundary

Because of the high levels of heterogeneity (75.5%) in the study populations and variations in the study designs, we used a random-effects model. By combining the findings from the 14 treatments across the 9 studies [29, 31–34, 37, 39–41], we found a significant reduction in fasting glucose levels following chitosan supplementation (SMD: − 0.39 mmol/L, 95% CI: − 0.62 to − 0.16) (Fig. 3). Because of the differences in dosage, duration of intervention, type of study, and age of study population, subgrouping was required for further analysis. Trial sequential analysis (TSA) of the pooled results of the effects of chitosan consumption on fasting glucose levels was performed. The cumulative sample size was over the required information size (RIS) of 7242 and cumulative Z-curve crossed both the trial sequential monitoring boundary and conventional boundary (Fig. 6a). We found no significant effect of chitosan intake on insulin levels by combining data from three available studies (four arms) [33, 39, 43] (SMD: − 0.20 pmol/L, 95% CI: − 0.64 to 0.24) (Fig. 4). For TSA, the cumulative Z-curve was crossed conventional boundary but not crossed the trial sequential monitoring boundary. Therefore, insufficient and inconclusive evidence was obtained (Fig. 6b). The intake of chitosan reduced HbA1c levels in patients included in the three available studies (four arms) [33, 41, 42] (SMD: -1.10; 95% CI: − 2.15 to − 0.06) (Fig. 5). TSA of the pooled result of HbA1c showed that the cumulative sample size was over the RIS of 2810 and cumulative Z-curve crossed both the trial sequential monitoring boundary and conventional boundary (Fig. 6c).

### Subgroup analysis

Subgroup analysis according to dose revealed a significant reduction in fasting glucose levels in the subjects administered 1.6–3 g chitosan per day (SMD: − 0.42 mmol/L; 95% CI: − 0.57 to − 0.26) and < 1.6 g chitosan per day (SMD: − 0.29 mmol/L; 95% CI: − 0.51 to − 0.07). Moreover, compared with studies with ≤13 weeks of supplementation (SMD: − 0.18 mmol/L; 95% CI: − 0.33 to 0.03), those with > 13 weeks of supplementation significantly reduced glucose levels (SMD: − 0.48 mmol/L; 95% CI: − 0.64 to − 0.32). Both the < 50 age group and ≥ 50 age group showed that chitosan supplementation could reduce blood glucose levels. Parallel design trials and cross-over design trials reported significantly reduced fasting glucose levels. When stratified by study population, chitosan supplementation among subjects with diabetes (SMD: − 0.66 mmol/L; 95% CI: − 0.96 to − 0.36) and who were overweight/obese (SMD: − 0.39 mmol/L; 95% CI: − 0.55 to − 0.24) resulted in a significant reduction in fasting glucose levels, whereas there was no effect among those with dyslipidemia (SMD: − 0.12 mmol/L; 95% CI: − 0.30 to − 0.06) (Table 2).

**Table 2:** Relationship between chitosan and glucose based on subgroup analysis

Variable	Chitosan group (n)/Control group (n)	No. of treatment arms	Pooled SMD(mmol/L)	95% CI	Z-value	***P***-value	I-squared (%)
**subgroups**							
**Dose of chitosan (g/d)**							
< 1.6	137/135	4	−0.29	−0.51 - -0.07	2.62	0.009	86.60
1.6–3	336/332	8	−0.42	− 0.57 - -0.26	5.28	0.000	74.20
≥3	151/151	2	−0.16	−0.39- 0.06	1.40	0.160	0.00
**Intervention duration (weeks)**							
> 13	315/310	5	−0.48	−0.64 - -0.32	5.90	0.000	81.20
≤ 13	341/343	9	−0.18	−0.33 - -0.03	2.35	0.019	67.40
**Study design**							
Parallel	460/460	10	−0.35	−0.48 - -0.22	5.20	0.000	70.10
Crossover	196/193	4	−0.27	−0.47 - -0.06	2.58	0.010	86.60
**Age**							
< 50	516/507	9	−0.34	−0.48 - -0.02	2.10	0.036	81.80
≥ 50	140/146	4	−0.25	−0.47 - -0.22	5.42	0.000	0.70
**Study population**							
Diabetes	94/95	3	−0.66	−0.96 - -0.36	4.29	0.000	87.40
Overweight/Obese	320/314	6	−0.39	−0.55 - -0.24	4.86	0.000	80.00
Dyslipidemia	242/244	5	−0.12	− 0.30 - 0.06	1.31	0.189	0.00

## Discussion

The overall results of our meta-analysis support the finding that chitosan supplementation could reduce glucose and HbA1c levels, whereas it has no effects on insulin levels. Because the heterogeneity among studies was high, subgroup analysis was performed, revealing a significant reduction in glucose according to the chitosan dose (1.6–3 g/day) and duration (> 13 weeks). Although the range of chitosan lowering of the glycemic index was not as obvious as that of first-line diabetes drugs such as glimepiride/metformin [54], it can be used as a long-term dietary fiber supplement to reduce the blood glucose load and improve insulin sensitivity, thus affecting insulin-like growth factors [55, 56]. Dietary fiber has many health benefits and addresses the specific health problems of patients with MetS. In addition to hypoglycemic effects, dietary fiber has favorable effects on serum low-density lipoprotein-cholesterol, triglycerides [57], and blood pressure and helps with weight management [58]. Thus, patients with MetS should be urged to consume more fiber than that recommended for the general population. Our results showed that high-dose chitosan intake did not affect fasting glucose levels, and there was no evidence that high-dose chitosan had toxic effects or side effects unrelated to treatment; however, excessive dietary fiber may cause gastrointestinal discomfort and affect the absorption of protein, minerals, and some trace elements [59, 60].

Stratified analysis of the study populations showed that the effect of chitosan on blood glucose of diabetic patients is greater than that of overweight/obese subjects, but it has no in subjects with dyslipidemia. A possible explanation for this finding is that patients with dyslipidemia did not definitely have diabetes. Chitosan intake may not greatly affect the normal physiological regulation of blood glucose and insulin [37, 39]. Few studies have focused on the mechanism of chitosan oligosaccharides in regulating blood glucose levels in patients with diabetes. A study have shown that chitosan exerts its antidiabetic effect by inhibiting the expression of intestinal α-glucosidase, glucose transporters, and peroxisome proliferator-activated receptor γ [33]. In addition, Chitosan stimulates the secretion of glucagon-like peptide 1, which is a small peptide with insulin secretory effects and is produced largely in the gut and neurons located in the nucleus of the solitary tract in the brain stem [38]. Another study suggested that chitosan oligosaccharide prevents hyperglycemia by inhibiting intestinal glucose digestion and transport and enhances glucose uptake, at least in part, by up regulating PPARγ expression of adiponectin in adipocytes [61]. In addition, Chitosan has been shown to improve glycolipid metabolic disorders by inhibiting inflammation and up-regulating peroxisome proliferator-activated receptor gamma expression, including by inducing weight loss, reducing fasting blood glucose levels, restoring intraperitoneal glucose tolerance, inhibiting overexpression of pro-inflammatory cytokines, and regulating factors related to glycolipid metabolism [62, 63].

Our findings demonstrate that a hypoglycemic effect of chitosan both in younger and elderly individuals. The ability of chitosan to regulate blood glucose is not restricted by age. As the diagnoses of diabetes are increasing in younger individuals, this condition is no longer considered to affect only elderly individuals [64, 65]. Chitosan may be useful as a dietary supplement or healthcare product for all people with diabetes or prediabetes.

HbA1c reflects the long-term glycemic level and is less affected by the current health status [42]. Our study showed that long-term intake of chitosan can reduce HbA1c levels. Kim et al. [33] showed that HbA1c was decreased significantly after 12 weeks in the chitosan group, whereas it was increased in the placebo group. These results have practical applications because they demonstrate the long-term effect of dietary chitosan on glycemic control. Zhao et al. [41] showed that patients from the chitosan oligosaccharide group attained an HbA1c reduction of 2.5% more than in other groups.

Only three (four treatment arms) with fewer than 100 participants of the 11 studies included in their review showed no changes in insulin levels. A previous study confirmed that chitosan does not improve insulin levels [43]. However, animal experiments showed that chitosan can reverse insulin resistance and decrease blood glucose [66]. In addition, dietary fiber was shown to improve blood glucose levels without regulating insulin, which is likely associated with one of the main health benefits of dietary fiber in regulating gastrointestinal transport [67]. Indeed, animal experiments showed that chitosan may prevent hyperglycemia by inhibiting intestinal glucose digestion [68] and carbohydrate hydrolysis enzymes [69]. According to the results of sequential analysis, additional clinical research data are needed to confirm the role of chitosan in regulating blood glucose and its mechanism, particularly in regulating glycosylated hemoglobin and insulin.

Compared to previous studies, our study has several advantages. First, only two meta-analyses of chitosan have been performed, which evaluated body weight and lipid modification [25, 70]. This is the first meta-analysis to evaluate regulation of the glycemic index following supplementation with chitosan, making it the latest and most comprehensive study to date. Second, we used TSA to evaluate the influence of random error and repetitive testing, so as to increase the robustness of our findings. Finally, subgroup based on the participant characteristics and study design was performed to identify the impact of chitosan on various parameters in the meta-analysis. However, our study had some limitations. Glucose, insulin, and HbA1c indices were not the primary outcome indicators in most of the selected studies, and thus secondary outcomes of a “0” effect may not always have been presented in the results. Therefore, the possibility of publication bias could not be excluded. In addition, some other confounding factors were not considered, such as dietary habit and economic situation, because of the lack of data in the available studies.

## Conclusion

Our results provide evidence for glycemic regulation by supplementation with chitosan. Specifically, supplementation of chitosan can improve fasting blood glucose in diabetic and obese/overweight patients. Based on our results, further studies to determine the effectiveness of chitosan in managing glucose levels in patients who are overweight/obese and/or diabetic should be designed with durations longer than 13 weeks and with an intake dose of 1.6–3 g per day. However, there is still insufficient evidence to evaluate the effects of chitosan on insulin. In addition, the long-term benefits and risks of chitosan intake remain unknown. Therefore, to understand the long-term efficacy of chitosan on blood glucose, insulin, and HbA1c levels, studies with a large sample, good experimental design, and population-based approach are needed to provide reliable data.

## Supplementary Information

**Additional file 1.** Search Strategy.

## Data Availability

Please contact author for data requests.

## Abbreviations

CIs: Confidence intervals
SMD: Standardized mean difference
MeSH: Medical subject headings
RCTs: Randomized controlled trials
HbA1c: Hemoglobin A1c
PRISMA: Preferred Reporting Items for Systematic Reviews and Meta-Analyses
TSA: Trial sequential analysis
RIS: Required information size
