# Supplementary material for: Chitosan modifies glycemic levels in people with metabolic syndrome and related disorders: meta-analysis with trial sequential analysis
Source: Nutr J. 2020 Dec 1;19:130. doi: 10.1186/s12937-020-00647-4 (PMC7709411; doi:10.1186/s12937-020-00647-4)
Supplement: Supplementary file 1 — Additional file 1. Search Strategy. [file 12937_2020_647_MOESM1_ESM.docx]

**Search Strategy**

| Database | Search Strategy | Citationa found |
| --- | --- | --- |
| Pubmed | ("Chitosan"[Mesh]) OR ((chitin[Title/Abstract]) OR (poliglusam[Title/Abstract]) OR(Chitosan oligosaccharide [Title/Abstract]) OR( Polyglucosamine[Title/Abstract])) | Total: 29608 Clinical trial: 190 |
| Embase | chitosan:ab,ti OR chitin:ab,ti OR poliglusam:ab,ti OR polyglucosamine:ab,ti OR 'chitosan oligosaccharide':ab,ti | Total: 44662 Clinical ariticle: 313 |
| Cochrance | #1 MeSH descriptor: Chitosan explode all trees  #2 (Chitosan oligosaccharide):ti,ab,kw OR (Polyglucosamine):ti,ab,kw | #1 AND #2  Total: 173  Trials: 172 |
